# Incentivizing optimal risk map use for *Triatoma infestans* surveillance in urban environments

**DOI:** 10.1371/journal.pgph.0000145

**Published:** 2022-08-03

**Authors:** Claudia Arevalo-Nieto, Justin Sheen, Gian Franco Condori-Luna, Carlos Condori-Pino, Julianna Shinnick, Jennifer K. Peterson, Ricardo Castillo-Neyra, Michael Z. Levy

**Affiliations:** 1 Zoonotic Disease Research Laboratory, One Health Unit, School of Public Health and Administration, Universidad Peruana Cayetano Heredia, Arequipa, Perú; 2 Department of Biostatistics, Epidemiology and Informatics, University of Pennsylvania, Philadelphia, Pennsylvania, United States of America; CSIR-Indian Institute of Chemcial Technology, INDIA

## Abstract

In Arequipa, Peru, a large-scale vector control campaign has successfully reduced urban infestations of the Chagas disease vector, *Triatoma infestans*. In addition to preventing new infections with *Trypanosoma cruzi* (etiological agent of Chagas disease), the campaign produced a wealth of information about the distribution and density of vector infestations. We used these data to create vector infestation risk maps for the city in order to target the last few remaining infestations, which are unevenly distributed and difficult to pinpoint. Our maps, which are provided on a mobile app, display color-coded, individual house-level estimates of *T*. *infestans* infestation risk. Entomologic surveillance personnel can use the maps to select homes to inspect based on estimated risk of infestation, as well as keep track of which parts of a given neighborhood they have inspected to ensure even surveillance throughout the zone. However, the question then becomes, how do we encourage surveillance personnel to actually use these two functionalities of the risk map? As such, we carried out a series of rolling trials to test different incentive schemes designed to encourage the following two behaviors by entomologic surveillance personnel in Arequipa: (i) preferential inspections of homes shown as high risk on the maps, and (ii) even surveillance across the geographical distribution of a given area, which we term, ‘spatial coverage.’ These two behaviors together constituted what we termed, ‘optimal map use.’ We found that several incentives resulted in one of the two target behaviors, but just one incentive scheme based on the game of poker resulted in optimal map use. This poker-based incentive structure may be well-suited to improve entomological surveillance activities and other complex multi-objective tasks.

## Introduction

Vector-borne diseases kill at least 700,000 people each year [1]. A common method for interrupting vector-borne disease (VBD) transmission is to target vector populations, especially blood feeding insects, in human-dominated areas, often through large-scale vector control campaigns. These efforts have successfully reduced incidence of VBDs such as malaria and Chagas disease [2–4], and in the process have also produced a wealth of information about vector distribution and density [5]. Such data could be used to guide subsequent surveillance and control activities [6].

One avenue for utilizing vector control data is to incorporate them into risk maps. In recent years, risk map technology has advanced rapidly [7–11]; computational power now allows for precise inference over large areas [12–14], while mobile technology can bring up-to-date predictions directly to surveillance personnel in the field [10,15,16]. Nonetheless, achieving optimal use of the complex spatio-temporal information provided by risk maps still hinges on the behavior of the technicians tasked with the job. Simply generating and delivering a map does not ensure its appropriate use [10], which can require the end users to be cognizant of multiple types of information at once, in addition to any other behavioral changes involved when implementing a new tool at work.

Such is the case for the mobile app, VectorPoint, which provides risk maps for domestic infestations of the Chagas disease vector *Triatoma infestans* in Arequipa, Peru (pop: ~1.3 million). Chagas disease is a vector-borne infection caused by the parasite *Trypanosoma cruzi*. The disease is chronic if left untreated, leading to serious cardiac, gastrointestinal, and/or peripheral nervous system morbidity in about 30% of those infected [17].

Over the past two decades, a large-scale insecticide campaign in Arequipa has significantly reduced the number of buildings in the city with Chagas disease vector infestations, resulting in infrequent infestations of households (there are no wild vectors in the region) that are sporadically distributed across the urban landscape. At the height of the campaign, over 30 dedicated field personnel worked on vector control. However, that number has dropped with the prevalence of infestation and presently there are no full-time staff dedicated to Chagas disease vector control. Inspections for vector infestation are conducted by surveillance personnel who are responsible for a variety of disease prevention activities, in addition to routine laboratory work.

Due to these constraints in time and resources, only a small percentage (<20%) of houses in each district are inspected for vector infestation each year. These houses are often selected based on convenience, which can result in large swaths of uninspected houses. The risk maps in the VectorPoint app were created as an attempt to alleviate this issue by providing surveillance personnel with the opportunity to make evidence-based decisions about which houses to inspect for *T*. *infestans* in the challenging post-vector control campaign scenario. Personnel can use the risk map to select houses to inspect based on the house’s estimated risk of infestation, as well as keep track of which houses they have inspected in a given area to ensure that they surveil evenly throughout the area. However, the question then becomes, how to encourage surveillance personnel to use these two functionalities of the risk map, which we define as optimal map use.

Therefore, in this study, we asked if incentives could help to bridge the gap between risk map delivery and optimal map use by surveillance personnel. We operationalized optimal map use with the following two target behaviors by surveillance personnel: (1) preferential inspections of houses displayed in the risk maps as higher risk, and (2) inspecting houses that were distributed evenly throughout the area they were tasked with surveilling. We investigated this question through a series of rolling trials, and generated trial-specific hypotheses in an iterative manner, based on results from the previous trial. Specific hypotheses are presented below with the information for each trial.

## Methods

### Overview

We conducted a rolling series of four trials in which we evaluated infestation risk map use by vector control personnel (hereafter referred to as participants) under different incentive schemes. Each trial took place in a district of Arequipa, Peru that had a history of *T*. *infestans* infestation (described below).

### Ethics statement

All participants provided written informed consent to participate in the study. The study was approved by the institutional review boards of the University of Pennsylvania (protocol number 824603) and the Universidad Peruana Cayetano Heredia (protocol number 66427).

### Study sites

**Fig 1:**
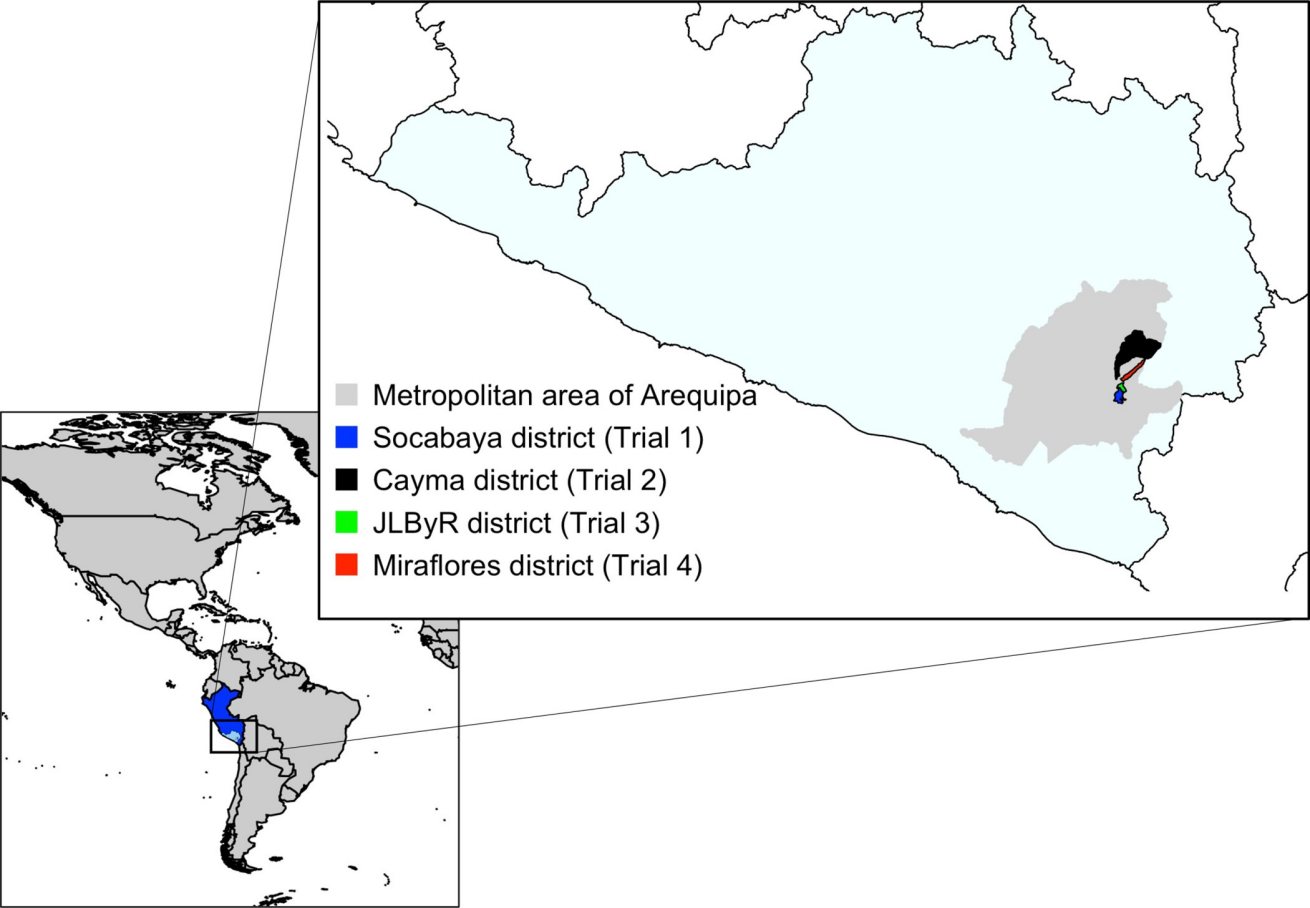
Map of study sites. Left: Western hemisphere with Peru shown in dark blue and the state of Arequipa in light blue. Right: Zoomed in map of the city of Arequipa (gray) and the study sites highlighted as described in the color-coded legend. The hemisphere map was created in R using the “maptools” package and the object “wrld_smpl,” which contains simplified world country polygons based on NASA data available on the NASA public Github page under ‘World-Wind-Java’ (https://github.com/nasa/World-Wind-Java/tree/master/WorldWind/testData/shapefiles). The Arequipa map was generated using our data.

Trials were conducted in four of Arequipa’s twenty-nine districts: the Socabaya district, the Cayma district, the Jose Luis Bustamante y Rivero (JLByR) district, and the Miraflores district (Fig 1). All districts had a population of at least 75,000 people, and a history of substantial *T*. *infestans* infestation during the insecticide treatment phase of the vector control campaign carried out between 2007 and 2012. Further demographic and historical information for each district are provided in the supplementary materials (S1 Text).

### Experimental design

**Fig 2:**
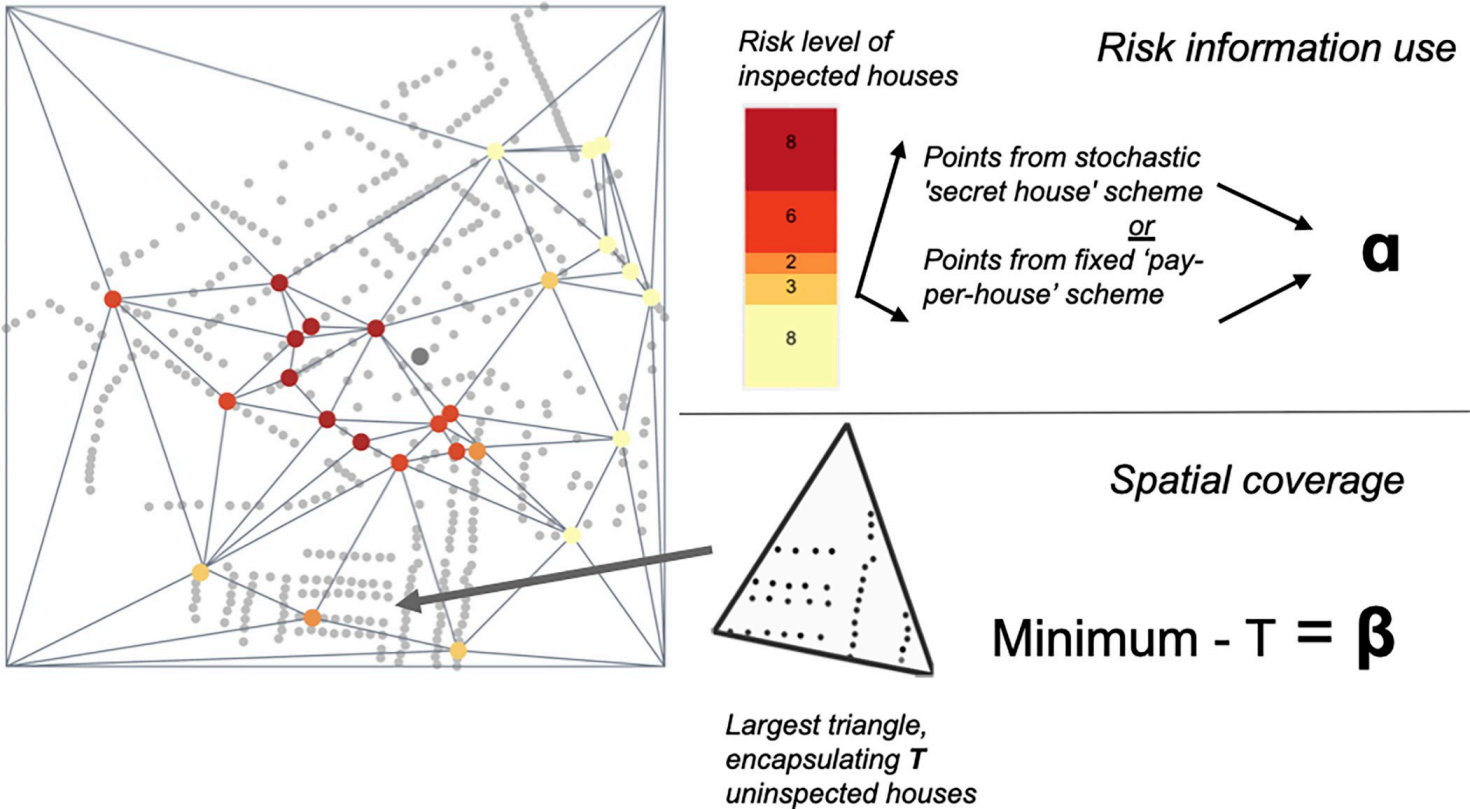
Representation of risk-based and spatial incentives. Left: Map displaying a search area of N households, each represented by a dot that is colored one of five colors representing relative risk of vector infestation. Top right: ‘**ɑ’** represents the reward for risk information use, which was earned in a stochastic or fixed incentive scheme. Bottom right: **β** represents the reward for spatial coverage, which is calculated by subtracting the maximum number of uninspected houses bounded by Delaunay triangles formed between the inspected houses, T, from the minimum expected coverage (N *.05). In the first three trials, the total reward was a weighted average of **ɑ** and **β**. In the final trial, values of **ɑ** and **β** were added together to form hierarchical ’poker hands’.

**Fig 3:**
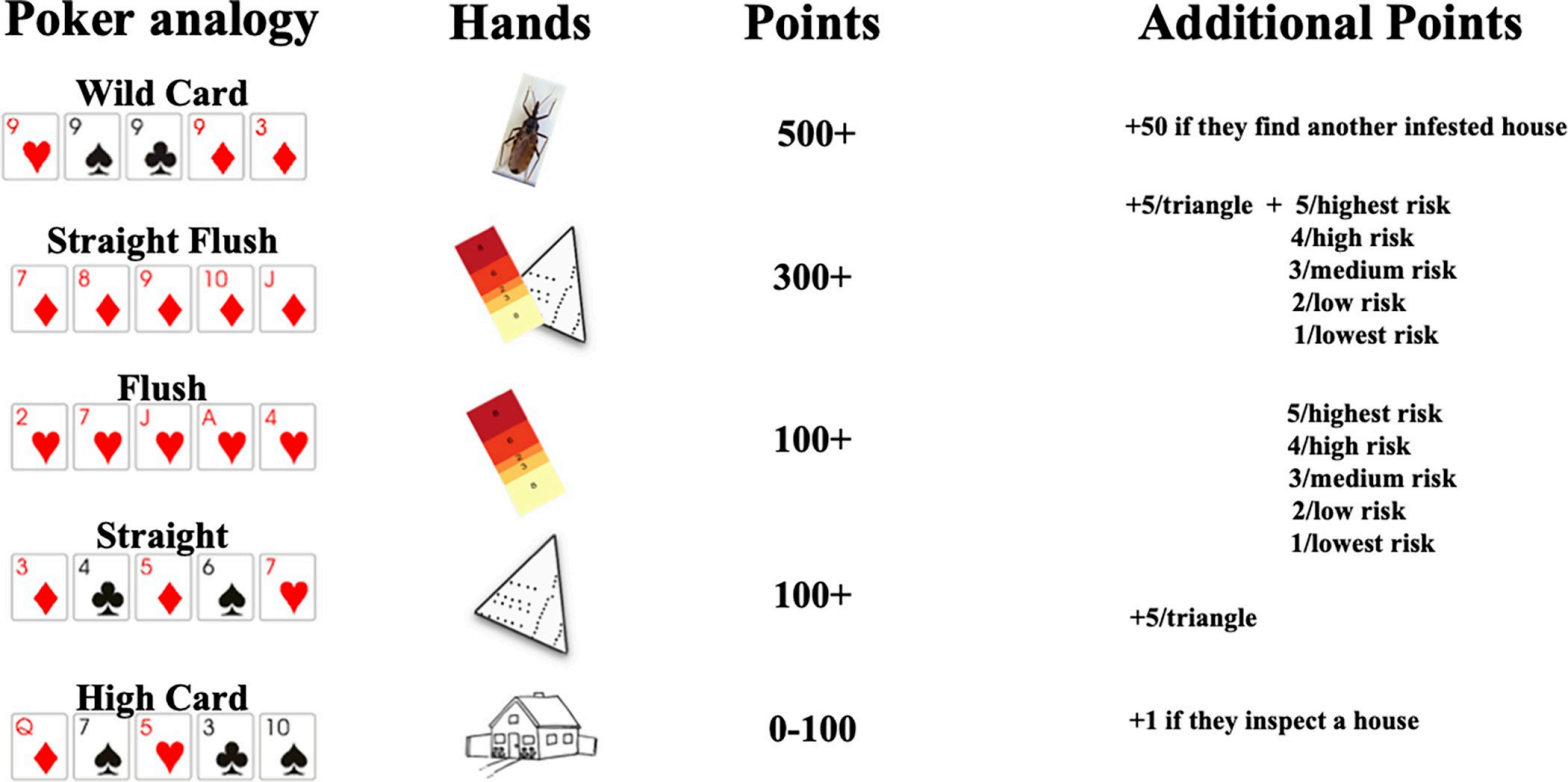
Poker incentive scheme. Column 1: Poker hands corresponding to each goal (i.e., different ways to earn points). Column 2: Images represent goals that participants could choose to achieve: Infested house detection (bug image), higher risk house inspections (five-color risk quintile image), and good spatial coverage (map image). The house image represents inspections regardless of house risk level or spatial coverage. Column 3: Base number of points for each hand. Column 4: Additional points (also referred to as ‘bonus points’) were awarded when participants exceeded the goals shown in the second column (details can be found in the poker hand descriptions in the text preceding the figure).

**Fig 4:**
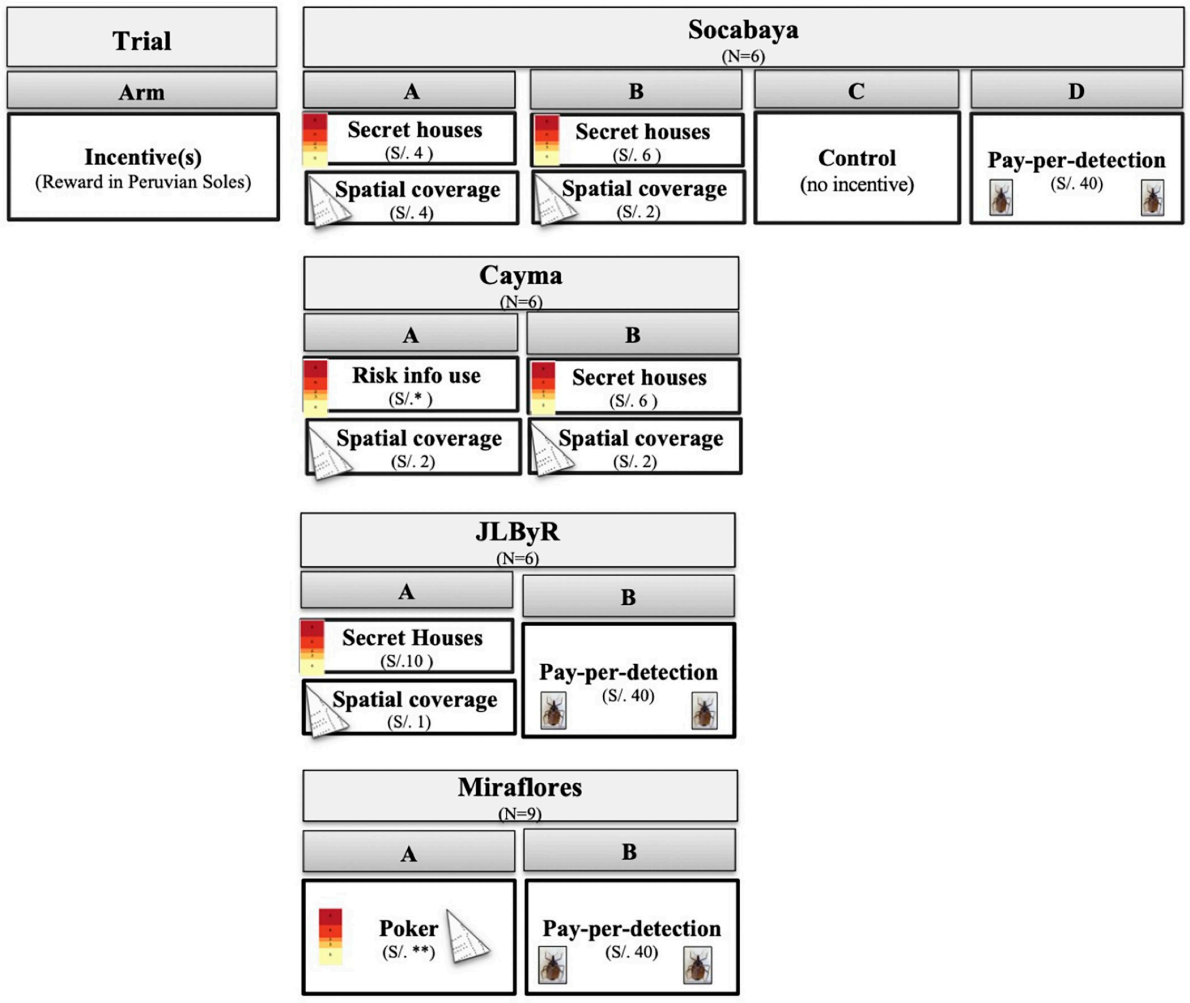
Experimental design overview. *Reward varied by house risk level. **Poker rewards were accrued as points that could be redeemed for vacation time. See Fig 3 for a full description of the poker rewards.

Each of the four trials had at least two arms in which we tested different incentive schemes. Trials followed a crossover design in which participants were compared to themselves across arms of the same trial [18]. Participants were assigned to a trial arm for five days (Mon-Fri), working for approximately five hours per day. For logistical reasons (vacations), some individuals did not participate in every trial. Six individuals participated in the first three trials, and nine in the final trial. Figs 2 and 3 provide incentive scheme overviews while Fig 4 illustrates the full experimental design.

In each trial arm, a participant was assigned to surveil a new ‘search zone,’ which were contiguous areas within one of the four districts that contained 400–600 households. This number of households is similar to that which a vector control technician might surveil in a given week. The boundaries of the zones were artificial, (as are the politically defined districts in which they are located), but they generally followed natural features of the landscape. Each search zone was included in the study only once. We randomized the assignment of search zones and the order in which each arm was carried out.

### VectorPoint app

Risk maps for vector infestation were provided on smart phones using our cloud-based mobile app called VectorPoint (described in detail in [10,19]). Briefly, the app provides risk maps based on current and historical data in addition to a data entry form that is used to record data from each home inspection, after which the data is sent to the data base and incorporated into the next run of the model (see below). Each participant had at least one month of experience using the VectorPoint app prior to the study.

### Risk maps

Risk maps displayed a given search zone in which each household in the zone was displayed as a dot that was colored one of five colors. Each of the five colors represented the risk of that household being infested with *T*. *infestans* relative to all other houses in the same search zone (Fig 2). Risk estimates displayed in the map were generated using infestation data from the Arequipa vector control campaign run in a longitudinal, spatio-temporal model described previously [10,19]. The model was run every evening to incorporate new information collected during inspections that day. To avoid any influence from vector surveillance activities in nearby areas, we ran the model on each search zone individually. Risk maps were updated and synched with the app each morning for participant use.

### Measuring optimal map use: Quantifying risk information use and spatial coverage

We measured risk information use by calculating the average risk level (ranging from one to five) of the houses inspected by each participant during each trial arm (i.e., one week) (see Box 1: Key Terms). We quantified spatial coverage of the search zone using a new functionality in the VectorPoint app that draws lines between inspected houses and divides the uninspected spaces into triangles (see Box 1: Key Terms). Every time a new house was inspected, the house became a vertex in a set of Delaunay triangles [20]. New triangles were formed in the app in real time, and were immediately visible to the participants, allowing them to monitor their spatial coverage. We used the number of uninspected houses in the largest triangle as the metric with which we evaluated spatial coverage (Fig 2), and this was also calculated for each participant at the end of each trial arm.

**Box 1: Key terms**

**Risk information use:** Preferential inspection of houses that are displayed in the risk maps as red or dark red, which represent the top two infestation risk quintiles (‘highest’ and ‘high,’ respectively).

**Spatial coverage:** The degree to which a search zone is evenly searched across its entire geographic area. The goal of spatial coverage is to avoid large swaths of inspected houses in any given area.

### Incentive structures

Each incentive structure was designed to encourage participants to use the household-level infestation risk information presented in the risk map while also displaying good spatial coverage of their search zone. We refer to this as our ‘dual objective,’ which we represent visually in Fig 2. Rewards were rewarded in Peruvian soles. One Peruvian sol is equivalent to approximately $0.28 USD.

- **Fixed risk information utilization:** Under this incentive scheme, one of five payment amounts was awarded to participants for each house they inspected. The five payment amounts were positively correlated with the five risk levels (as shown by five colors in the risk map) in order to encourage participants to utilize the household level risk information presented in maps.
- **Spatial coverage:** Under the spatial coverage incentive, participants were rewarded for surveilling evenly across the total area of their search zone, quantified as described above. A minimum expected coverage was established as 5% of the total number of houses in each search zone. As a reminder, the inspected houses formed vertices of a set of Delauney triangles [20]. The largest of these triangles—which represents the biggest ’hole’ in the search area, was identified (Fig 2). If the largest of the triangles contained fewer houses than the minimum expected coverage, a reward was given. The size of the reward increased with each subsequent decrease in the number of households situated in the largest triangle.
- **Secret houses:** With this incentive, we aimed to encourage participants to use the risk information in the maps by providing rewards for inspecting certain ‘secret’ houses that were not revealed to participants ahead of time. Participants were informed that houses with a higher infestation risk level had a higher probability of being ‘secret’ houses. We chose secret houses using weighted random selection, in which each house was weighted proportionally to its estimated risk quintile. We informed participants at the end of each day if they had inspected a secret house. Monetary values awarded for inspecting a secret house differed by trial and arm.
- **Pay per detection:** In this incentive structure we aimed to motivate participants to adopt both target strategies by allocating large financial rewards for detecting and inspecting infested houses. The probability of finding an infested house in Arequipa is very low, so the larger reward was balanced by the lower expectation of a payout.
- **Poker incentive structure (Fig 3):** Under this incentive structure points were awarded for achieving the goals described below. When participants accumulated 500 or 1000 points, they could trade the points in for four hours or a day off work, respectively. The possible goals (‘poker hands’) were:
  ○ **High card (one point per home inspection):** A point was awarded for each home inspection carried out, regardless of spatial coverage achieved or house risk level. A home inspection was analogous to a poker hand that has no flush or straight, and is thus scored on its highest card.
  ○ **Straight (100 points + possible bonus points):** The next level up was a ‘straight,’ which describes a poker hand of five cards in sequence, but of different suits. In this case, spatial coverage was analogous to card sequence; a ‘straight’ was achieved when the number of houses in the largest Delaunay triangle was less than 5% of the total number of houses in the search zone. Once the minimum spatial coverage requirement for the straight was achieved, bonus points were awarded; bonus points were calculated by first multiplying the difference between 5% of the number of houses in the search zone and the number of houses in the largest Delaunay triangle by five.
  ○ **Flush (100 points + possible bonus points):** A ‘flush’ refers to a poker hand with five cards of the same suit in no sequential order. In our poker incentive scheme, a flush was achieved when the mean risk level of all houses inspected in a week was at least four (out of five possible levels/quintiles). Once the minimum requirement for the flush was met, each inspection garnered one to five additional points corresponding to house risk level. For example, five additional points were awarded for each inspection of a highest risk house, four points for second highest risk level, and so on for all five risk levels.
  ○ **Straight flush (300 points + possible bonus points):** A ‘straight flush’ is a poker hand with five cards of the same suit (the flush) in sequential order (the straight). Hence, a straight flush was achieved under the poker incentive scheme by inspecting houses with a mean risk level of four *and* spatial coverage over 5%. The number of bonus points awarded for a straight flush was the sum of the additional points that would be calculated for a straight and a flush, as described above.
  ○ **Wild card (500 points + possible bonus points):** Detecting infestations in a low-prevalence situation involves some combination of strategy and luck. We draw the analogy to using a wildcard—which, in some versions of poker, confers great advantages to the player who draws it. In our poker incentive scheme, participants scored a ‘wild card’ by detecting an infested house. In addition, one bonus point was awarded for each house inspection, regardless of risk level.

### Statistical analyses

All statistical tests were carried out in the R statistical computing environment [21]. Given the ordinal nature of the outcome variable for risk information use, we used a proportional odds logistic regression (POLR function from the MASS Package [22]) to compare the number of houses that participants inspected in each risk quintile under the different arms of each trial. For analyses of spatial coverage in the trials with two arms (Cayma, JLByR and Miraflores trials, described below), we performed a paired t-test for each trial in which we compared spatial coverage between arms. To analyze spatial coverage in the Socabaya trial, which had three experimental arms and one control arm, we carried out a paired t-test comparing spatial coverage in each of the three experimental arms to that of the control arm.

## Results

For ease of comprehension, we preface the results for each trial with the hypothesis tested in the trial, as well as the trial set up. Table 1 is provided as a quick reference for the incentive scheme descriptions, and Fig 4 provides an overview of all trials. Trial names reflect the district in which they were carried out. Data for each participant are available in S1 and S2 Tables and S1 Fig. All results are summarized in Table 2. For each district, we report the number of houses visited and the number of houses inspected. A ’visit’ refers to when a technician goes to a house and requests permission to perform an inspection. If the house is inspected, then it is considered as such. If no one is home, or if the homeowner does not allow the inspection, the house is considered ’visited’ but not inspected. Only houses that were inspected were included in our incentive analyses.

**Table 1:** Overview of the incentive schemes tested in this study. Detailed information for each scheme is found in the methods section and in Figs 2–4.

Incentive	Description
Fixed risk information utilization	Participants rewarded for inspecting houses with a fixed value price based on the risk.
Secret houses	Participants rewarded for inspecting houses designated as ‘secret houses’ using random selection weighted toward higher risk houses
Spatial coverage	Participants rewarded for decreasing Delaunay triangle size beyond a minimum threshold.
Pay-per-detection	Participants rewarded for finding an infested house
Poker scheme	Participants rewarded for achieving tasks named for different poker schemes

**Table 2:** Results overview.

Trial	Arm	Incentive	Payout	Reward^1^	Behavior change
Socabaya	A	Secret houses	Stochastic	4 soles	None
		Spatial coverage	Fixed	4 soles	Increased spatial coverage**
	B	Secret Houses	Stochastic	6 soles	None
		Spatial coverage	Fixed	2 soles	Increased spatial coverage***
	C	None (control)	n/a	n/a	None
	D	Pay per detection	Stochastic	40 soles	None
Cayma	A	Spatial coverage	Fixed	2 soles	None
		Risk information use	Fixed	^	Increased risk information use*
	B	Spatial coverage	Fixed	2 soles	None
		Secret Houses	Stochastic	6 soles	None
JLByR^2^	A	Secret Houses	Stochastic	10 soles	None
		Spatial coverage	Fixed	1 sol	None
	B	Pay per detection	Stochastic	40 soles	None
Miraflores	A	Poker	Fixed	Vacation time (accrued in points)	Increased spatial coverage* AND Increased risk information use***
	B	Pay per detection	Stochastic	½ day off work	None

^1^One sol = $0.28 USD.

^2^ Jose Luis Bustamante y Rivero.

*Calculated by dividing 0.1,0.2,0.3,0.4, or 0.5 soles by a denominator of 6 soles. Significant difference (paired t-test or POLR model).

*p < 0.05

**p < 0.01

***p < 0.001.

### Trial one: Socabaya

#### Hypothesis

In this trial, our hypothesis was generated based on two previous pilot studies in which we observed that participants focused more on triangulation (spatial coverage) than using risk information. Thus, in this trial we tested the hypothesis that a higher reward amount for risk information use than for spatial coverage would increase risk information use.

#### Set up

This trial contained six participants and four arms (A-D). We tested three incentive schemes: secret houses (aimed to increase risk information use), spatial coverage, and pay per detection. Arm A awarded equal amounts (four soles) for spatial coverage and risk information use, while arm B awarded more for risk information use (six soles) than for spatial coverage (two soles). Arm C did not have any incentives, and served as the control to which Arms A, B and D were tested, and arm D had just one incentive, pay per detection.

#### Outcomes

**Fig 5:**
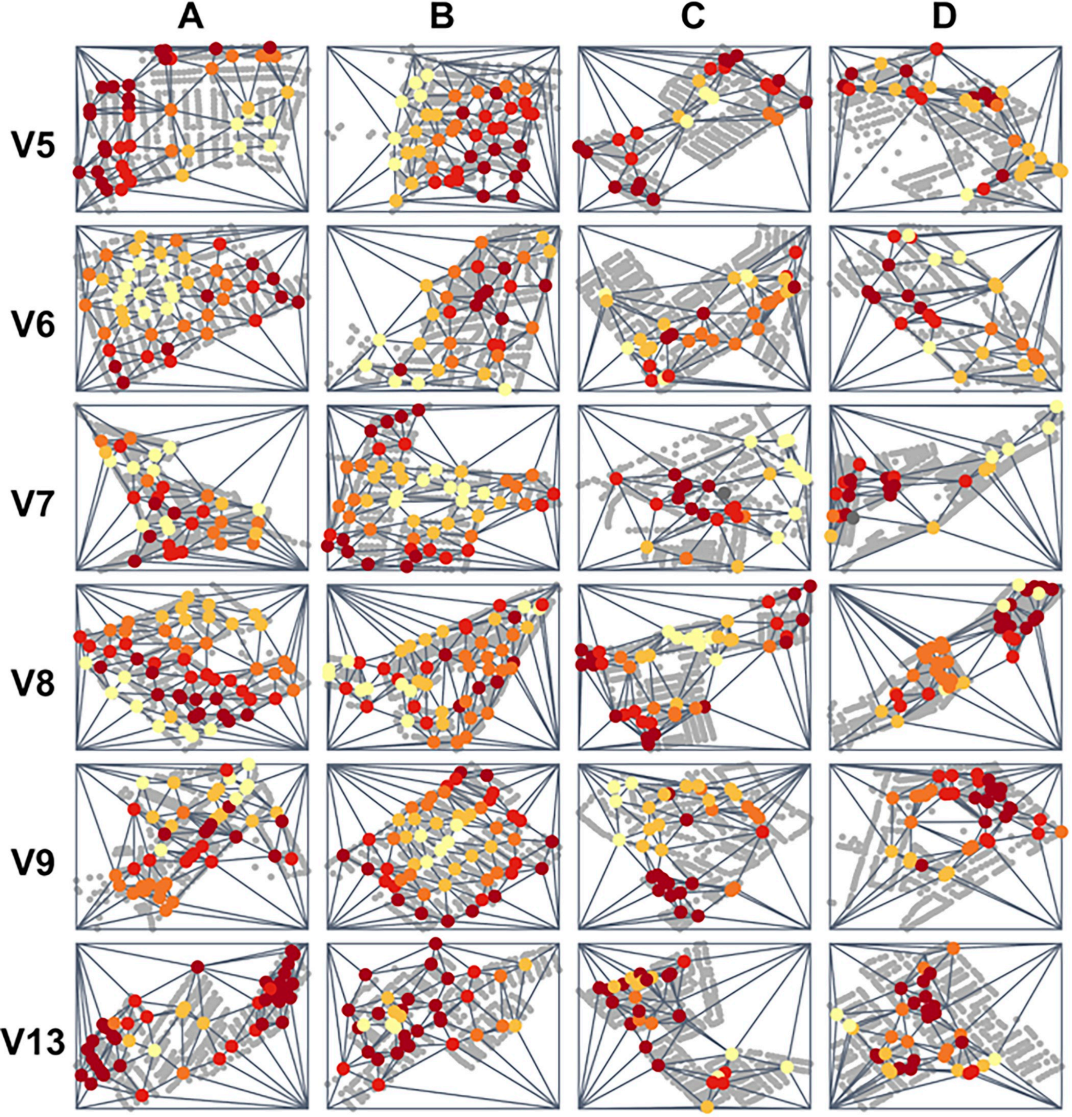
Spatial coverage maps for each participant in the Socabaya trial. Rows represent participants (represented by ‘V[number]’), columns represent trial arms. Incentives used in each arm were: (A) Fixed incentive for spatial coverage and a stochastic incentive for inspecting higher risk houses; (B) Fixed incentive for spatial coverage and an increased payout of the stochastic incentive for inspecting higher risk houses; (C) no incentives (control arm); and (D) pay per detection. Spatial coverage is represented by Delaunay triangulation in which triangles are formed connecting inspected houses (colored dots represent the inspected houses by the infestation risk shown as a gradient from yellow (lowest quintile) to dark red (highest quintile). Arms A and B both had significantly higher spatial coverage than Arms C and D (paired t-test, p < 0.01, p < 0.001, respectively).

Over the course of four weeks, six participants visited a total of 2,824 houses and conducted 965 inspections in 24 search zones. Financial incentives significantly improved spatial coverage in arms A and B (Fig 5; Arm A: paired t-test, t = -5.4155, p < 0.01; Arm B: paired t-test, t = -7.6817, p < 0.001) in comparison to the control arm. The average size of the largest triangle in Arms A and B, (the two arms in which spatial coverage was incentivized) was 18.0 uninspected houses (range: 10.0 to 26.0) and 15.7 uninspected houses (range: 10.0 to 25.0), respectively. In Arms C and D, (in which spatial coverage was not incentivized), the largest group of uninspected houses averaged 52.7 (range: 35.0 to 71.0) and 50.0 (range: 35.0–72.0), respectively. We found no significant differences in risk information use in any of the arms (A, B and D) when compared with the control arm (S1 Fig). The monetary value of the economic incentives awarded in this trial was an average of 10.1% of a participant’s salary (ranging from 4.0% to 13.0%).

#### Conclusion

A higher reward amount for risk information compared to spatial coverage did not increase risk information use. Spatial coverage was good regardless of the payout.

### Trial two: Cayma

#### Hypothesis

In this trial, we hypothesized that the relatively low use of risk information in the Socabaya trial might be due to the uncertainty involved in searching for secret houses, as compared to increasing spatial coverage. To test this hypothesis, we replaced the secret houses incentive with a fixed incentive for increasing risk information use.

#### Set up

The trial consisted of six participants and two arms (A and B). In arm A, we awarded a fixed incentive for risk information use (reward amount described below), and two soles for spatial coverage. In arm B, we used the same incentives as in Arm B of the previous trial: secret houses (six soles) and spatial coverage (two soles).

For the fixed risk utilization, five reward amounts (0.1, 0.2, 0.3, 0.4 and 0.5 soles) corresponding to the five house infestation risk levels (lowest, low, medium, high, and highest) were awarded, as calculated by:

$$\frac{0.5,\;0.4,\;0.3,\;0.2,\;\mathrm{or}\;0.1\;\mathrm{soles}}{6\;\mathrm{soles}}$$

Six soles were selected for the denominator to align with the secret houses incentive amount in arm B.

#### Outcomes

**Fig 6:**
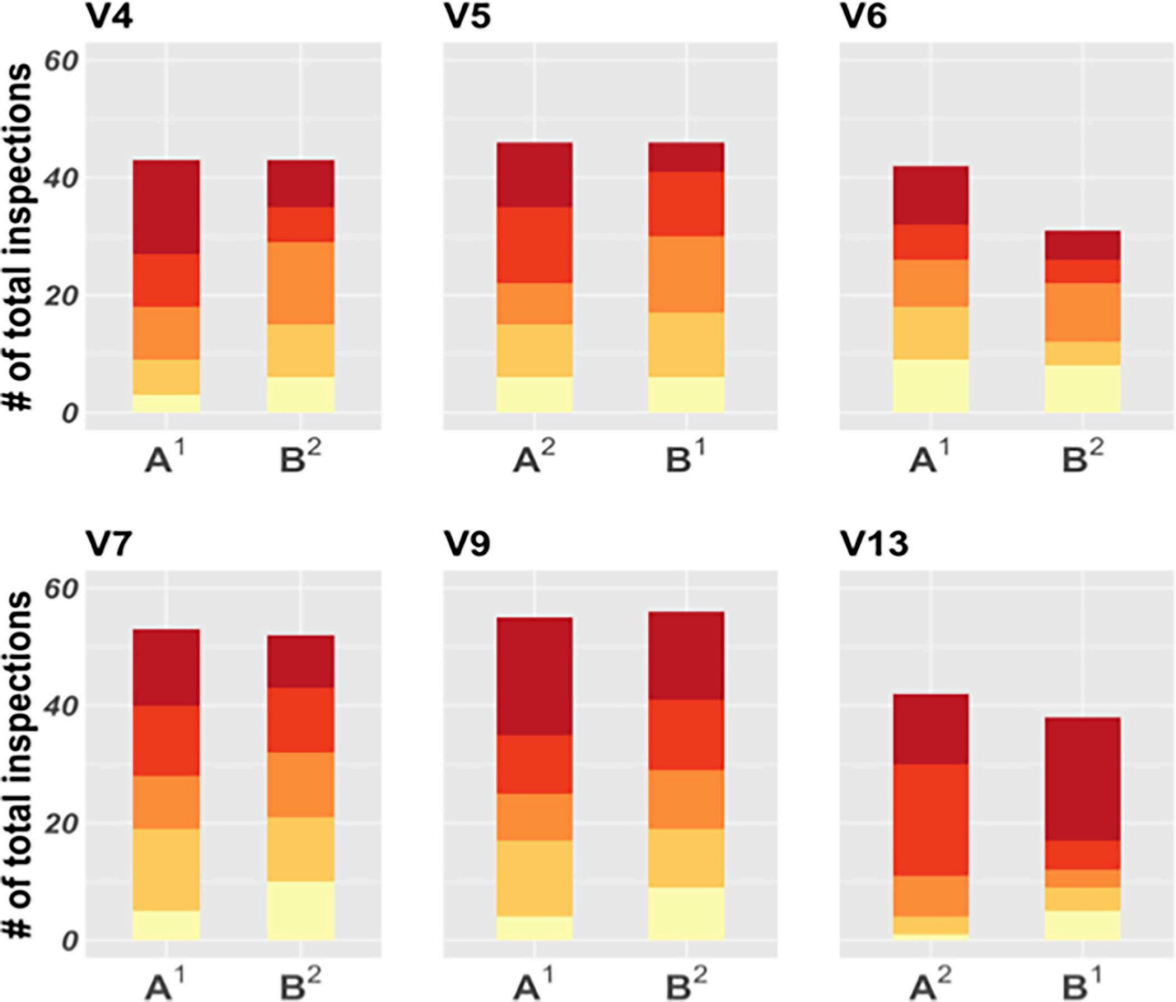
Infestation risk quintile distribution of households inspected by participants in the Cayma trial. Each set of two bars represents one participant, and each bar a study arm (A or B). A fixed incentive was used in arm A while the incentive in arm B was stochastic. Colors are ordered by risk quintile, going from the lowest (light yellow, bottom) to the highest (dark red, top). Arm A, (fixed incentive) had significantly higher risk information use than Arm B (stochastic incentive; POLR model, p < 0.02). Superscripts 1 and 2 in the x axis text indicate arm order.

The fixed payment significantly increased risk information use, (POLR model, OR 1.45, 95% CI [1.08–1.96], p < 0.02, Fig 6) compared to the stochastic payout scheme of the secret houses incentive. The average size of the largest triangle in arms A and B was 19.2 (range:10.0–44.0) and 17.8 (range: 9.0–29.0), respectively. There was no significant difference in spatial coverage between arms A and B. Payouts in arm A ranged from 1.0% to 6.5% of the participants’ monthly salaries (mean 4.8%), and arm B payouts averaged 5.0% of a participant’s salary (ranging from 0.5% to 6.5%).

#### Conclusion

The fixed incentive increased risk information use, but average risk level of inspected houses still fell below our goal of at least risk level four. Spatial coverage was good, with the largest triangle of uninspected houses being less than five percent of total homes in the search zone.

### Trial three: Jose Luis Bustamante y Rivero (JLByR)

#### Hypothesis

In this trial, we hypothesized that much higher payouts for inspecting higher-risk houses, as compared to spatial coverage payouts, would lead to increased risk information use. We returned to using a stochastic incentive to encourage risk information use, as we felt the approach was better understood by participants.

#### Set up

There were six participants and two arms in this trial. Again, we employed three incentive schemes: secret houses (aimed to increase risk information use), spatial coverage, and pay per detection. In arm A, we offered a payout of 10 soles for the secret houses incentive and one sol for increased spatial coverage. In arm B, we employed the pay-per-detection incentive.

#### Outcomes

**Fig 7:**
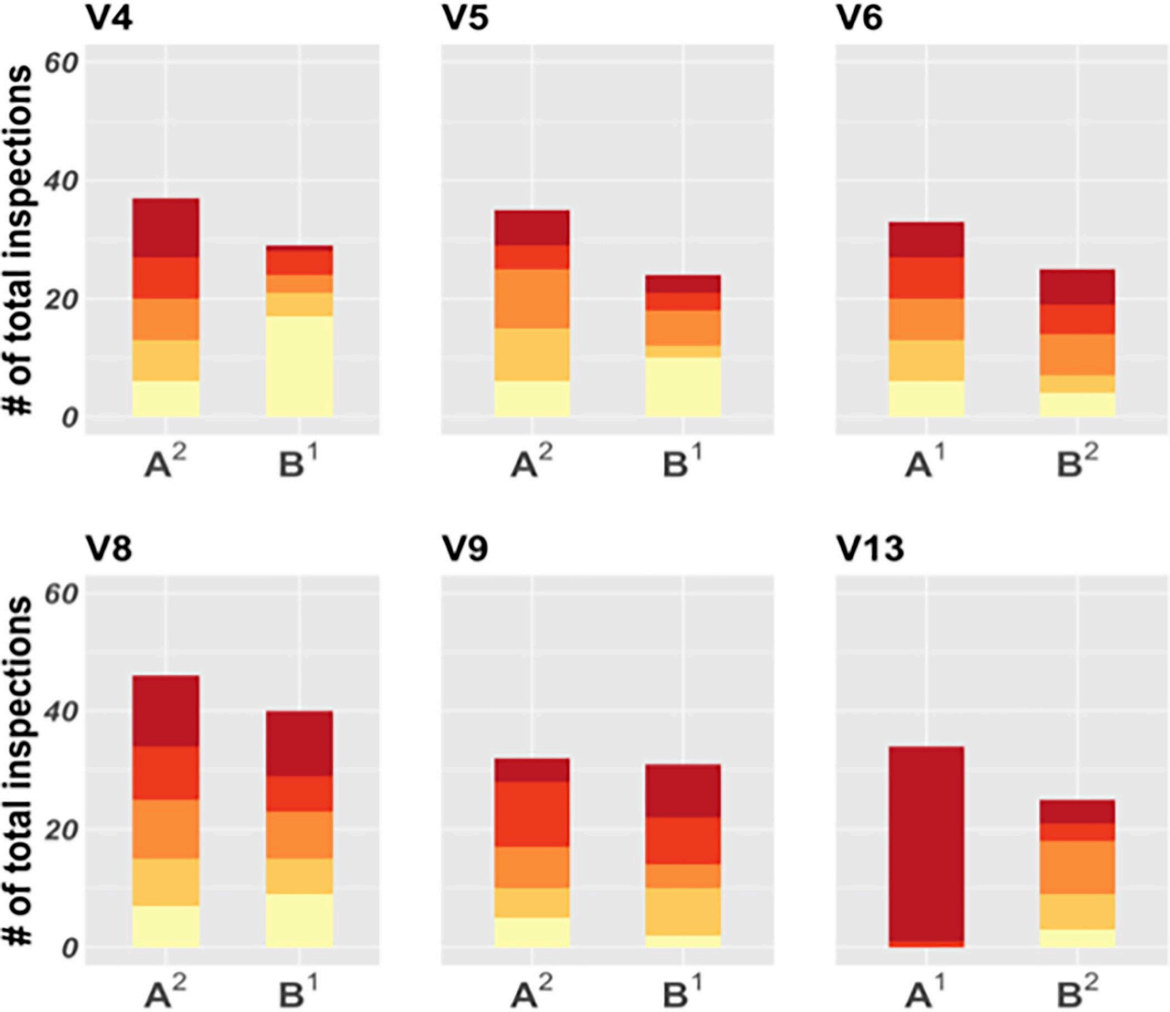
Distribution of household infestation risk quintile of households inspected by participants in the Jose Luis Bustamante y Rivero (JLByR) Trial. Each set of two bars represents one participant, and each bar, a study arm (A or B). Colors are ordered by risk quintile, going from the lowest (light yellow, bottom) to the highest (dark red, top). In arm A, the secret houses and spatial coverage incentives were used, while arm B employed only the pay-per-detection incentive. Superscripts 1 and 2 in the x axis text indicate arm order.

We observed divergent results among participants in this trial. In arm A, one participant carried out 95% of their inspections in houses in the highest risk category, and the remaining 5% of their inspections were carried out in houses in the second-highest risk category, Fig 7). This participant greatly increased their use of risk information relative to arm B, but at the expense of spatial coverage. The other five participants did not significantly increase their inspections of higher risk houses in Arm A when compared to Arm B (POLR, odds ratio (OR) = 1.35, 95% CI [0.92–1.98], p < 0.2). The average size of the largest triangle in arms A and B was 42.5 (range: 10.0–120.0) and 60.0 (range: 20.0–77.0), respectively. We did not find any significant difference in spatial coverage between Arm A and Arm B. The average award in arm A was 0.67% of participants’ salaries (ranging from 0 to 1.71%).

#### Conclusions

Greatly increasing the payout amount did not lead to an overall increase in risk information use by participants; in the single case where it did increase information use, spatial coverage was sacrificed. Better balanced incentives are needed to achieve the dual objective.

### Trial four: Miraflores

#### Hypothesis

Given our observation that participants tended to exhibit one target behavior but not both, we hypothesized that if the incentive payout increase could occur only when both target behaviors were carried out, we would achieve our dual objective of increased risk information use and increased spatial coverage.

#### Set up

This trial had nine participants and two arms. In arm A of this trial, we introduced a new incentive structure that was analogous to different hands of poker (described in detail in the Methods and in Fig 3). Rewards were given in the form of points that could be traded in for time off work, rather than monetary incentives. In arm B of this trial, we used the pay per detection incentive, with 500 points awarded for detecting an infested house.

#### Outcomes

Participants inspected a significantly greater number of high-risk houses (POLR, OR = 2.11, 95% CI [1.52–2.93], p < 0.001), and achieved better spatial coverage (paired t-test, t = 2.40, p < 0.05) when compared to the pay per detection arm. The largest group of uninspected houses averaged for poker arm and control arm contained 33.0 houses (range: 17.0–46.0) and 68.3 houses (range: 30.0–109.0), respectively). Two participants received half a day off (valued at 1.6% of their monthly salary) by earning 500 points (V8 and V9) under the poker arm. No participant received a reward under the points per detection arm.

#### Conclusion

**Fig 8:**
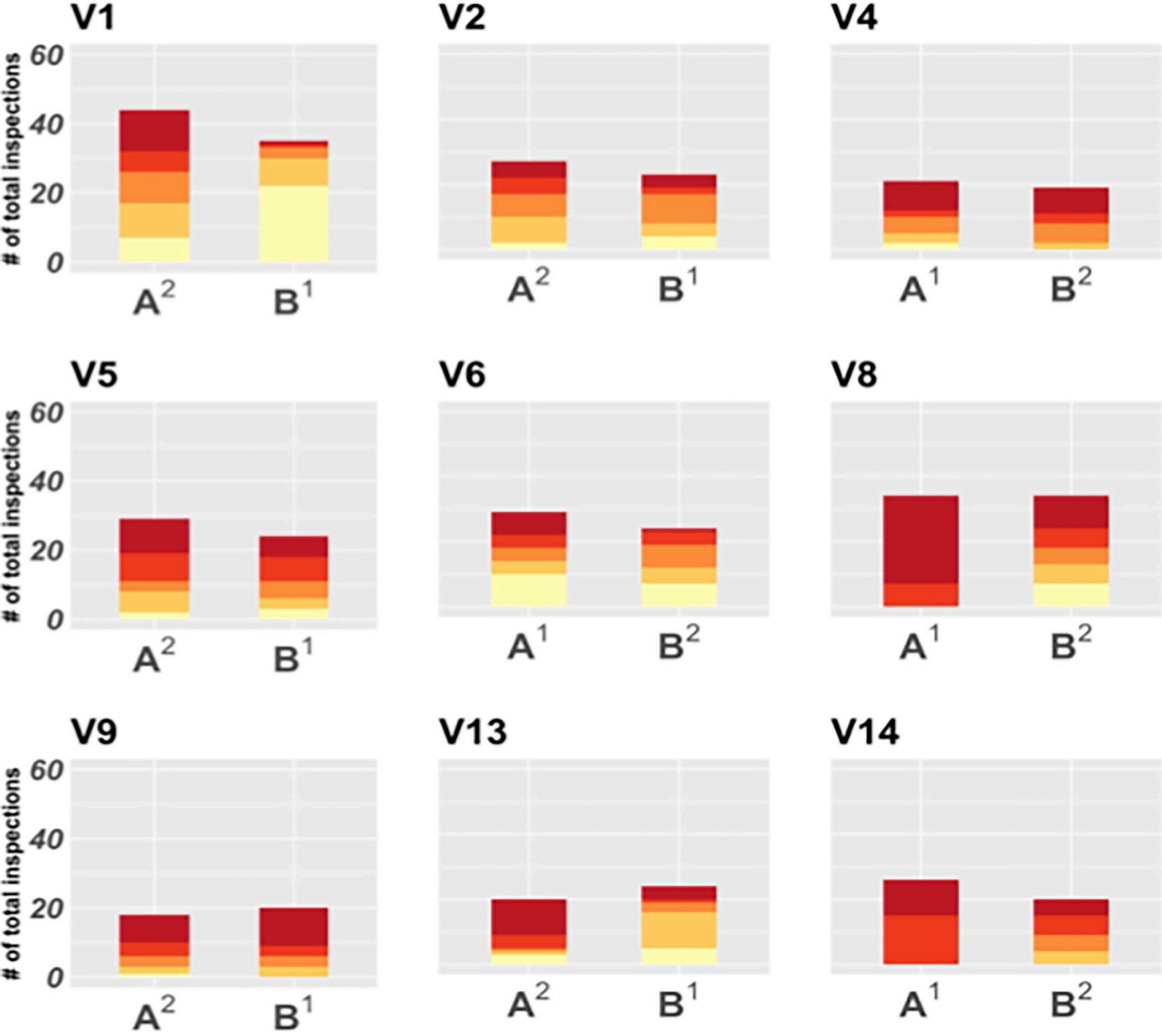
Distribution of household infestation risk quintile of households inspected by participants in the Miraflores Trial. Each set of two bars represents one participant, and each bar, a study arm (A and B). Colors are ordered by risk quintile, going from the lowest (light yellow, bottom) to the highest (dark red, top). Arm A was the poker incentive while arm B was pay per detection. The poker arm (A) had significantly different risk information use than arm B (POLR model, p < 0.001). Superscripts indicate arm order.

**Fig 9:**
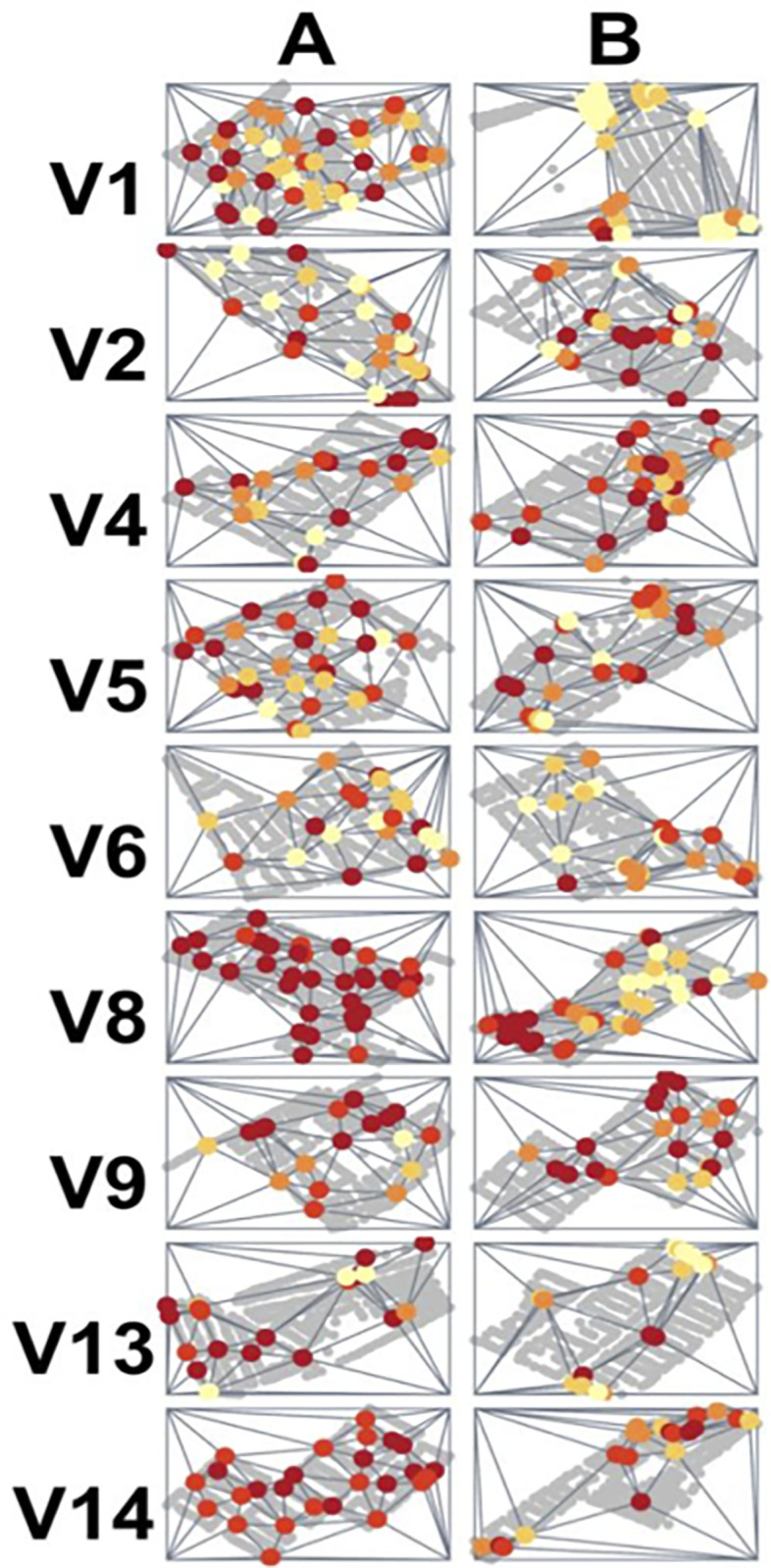
Spatial coverage maps for each participant in the Miraflores trial. Rows represent nine participants; columns represent trial arms. Incentives used in each arm were: A) poker incentive; and B) pay per detection. Spatial coverage is represented by Delaunay triangulation in which triangles are formed connecting inspected houses (colored dots represent the inspected houses by the infestation risk shown as a gradient from yellow (lowest quintile) to dark red (highest quintile). Arm A had significantly higher spatial coverage than Arm B (paired t-test, p < 0.05).

The poker incentive scheme resulted in achieving our dual objective of increasing risk information use (Fig 8) and spatial coverage of the search zone (Fig 9).

## Discussion

The World Health Organization calls surveillance a ’cornerstone’ of public health security and practice [23]; if it is a cornerstone, it is one that must be constantly shaped to different architectures of control agencies, and new epidemiologic scenarios [24,25]. Urban disease vectors present a unique surveillance challenge, especially when infestations are infrequent and/or control personnel reduced. In these cases, evidence-based tools such as risk maps can enhance field surveillance, especially if used to their full advantage. In this series of rolling trials, we evaluated infestation risk map use by entomologic surveillance technicians under different incentive schemes. We used incentives to encourage the adoption of two search strategies: maximal spatial coverage of the surveillance zone and preferential inspections of houses with higher infestation risks. We found that technicians adopted one of the search strategies under several of the incentive structures, but our dual objective was only achieved under the poker scheme.

In pursuit of the incentive structure that would achieve our dual objective, we observed that stochastic incentives (secret houses and pay-per-detection) did not successfully influence participant behavior, even with payout amounts that were higher than those associated with fixed incentives. A preference for guaranteed payouts over lotteries or variable micropayments has been observed in some prior studies [26–29], although many revolved around one-time executions of target behaviors [27–29], and the collective findings are variable [30]. Regardless, a preference for the known versus the unknown (ambiguity aversion [31]) is a widely-observed phenomenon [32], and it is fairly unsurprising that we observed this preference when incentivizing more complex behaviors.

Despite the success of fixed payments in incentivizing a single behavior, when we used weighted incentives to encourage both high spatial coverage and preferential inspections of higher risk houses, we failed. Participants apparently discovered that they could more easily maximize their earnings by focusing solely on the spatial triangulation. When incentives were more heavily weighted toward risk utilization, one inspector abandoned attempts to achieve high spatial coverage and focused solely on visiting higher risk houses. This outcome may be due to the arduous nature of the work itself- searching for infrequent and sporadically distributed vectors across a large city is tedious at best. The simplest route to the fastest payout may well have been the more salient option if intrinsic motivation was low from the beginning, which has been observed in health-related behaviors (reviewed in [33–35]), although less so in health workers specifically [36]. Weighted incentives, at least in our case, seem to be limited in their ability to encourage complex behaviors. Only when we introduced the poker incentive scheme did participants both preferentially inspect higher risk houses and maximize their spatial coverage. In the poker scheme, the payouts for achieving each objective were complementary- there was no way to earn a higher reward without achieving both objectives. For instance, to achieve the high scoring, “straight flush,” inspectors needed to both visit high risk houses and achieve substantial spatial coverage to receive a single fixed reward.

In epidemiologic surveillance, incentives or stipends have been used to increase data collection through new mobile tools, surveys, or disease testing, both at the individual/family level [37–40] and at the community level through Community Health Workers (CHWs; [41–43]. Many recent studies tested uptake of new mobile disease surveillance tools in low and middle income countries [40,41,43,44]. A handful of vector control studies have successfully incentivized citizen scientists using cash rewards for data collection [45,46], or by using paid, online crowd-sourcing to collect data [47]. Relatively few epidemiologic surveillance studies have attempted to use incentives to encourage more complex behaviors. In one notable study, salary incentives were used to increase uptake of a mobile, cloud-based tool for dengue surveillance by Public Health Inspectors in Sri Lanka. While spatial coverage was not encouraged with specific incentives, the authors observed that salary and bonus increases did result in a better spatial coverage [41].

The poker scheme worked well for entomologic surveillance. As it is an easily communicated means to encourage multi-objective tasks, it could be generalized to other surveillance scenarios, and perhaps much more broadly. The success of the Miraflores trial also suggests that non-monetary incentives may be a feasible option when incentivizing complex behaviors, which may be especially useful for public health departments, whose resources are often scarce [48]. In addition to potentially being more cost-effective than cash payouts, some studies have found that non-monetary incentives have unique benefits such as increasing employee investment in their organization [49], social reinforcement if other team members see the incentive received, and simply the enjoyment derived from looking forward to the incentive [50].

Our trial was short for practical reasons; we do not know if we would obtain the same results over the years or decades-long course of a vector surveillance campaign. The poker incentive was used during the last study in a rolling trial, so it is possible that technicians became more accustomed to responding to incentives as they participated in each sequential study. Furthermore, we have previously found that there is a great deal of heterogeneity in surveillance among technicians, whether measured by sensitivity [51] or productivity [10]. Given our relatively small sample size, individual differences could change in response to each incentive. Finally, we measured improvement in two domains: spatial coverage and risk level utilization. Although these metrics are used as surrogates for optimal infestation risk map use and effective surveillance, we do not know if improvement in both of these realms will lead to identifying more infested houses.

Eliminating disease vectors in cities involves familiar challenges: insecticide resistance, resistance to using insecticides in general, and resistance to changing the *status quo* [6,52]. In the post-spray phase of a largely successful vector control campaign, surveillance strategies must evolve and adopt new control methods that are suited to their post-spray scenario if they are to prevent vector re-emergence. Implementation of data-based tools is a feasible option, although real improvements will only occur with the buy-in of those doing the day-to-day work in the field. Using an incentive such as the poker scheme, that is customized to achieve specific program objectives, is one potentially feasible approach to bridging the gap between tool design and optimal tool use.

## Supporting information

S1 Fig Distribution of infestation risk quintile for the households inspected by participants in the Socabaya Trial. Each set of four bars represents one participant, and each bar a study arm (A-D). Colors are ordered by risk quintile, going from the lowest (light yellow, bottom) to the highest (dark red, top). Superscripts above each arm name (A-D) in the x axis text indicate arm order.
(TIFF)

S1 Table Number of uninspected houses in the largest triangle. (DOCX)

S2 Table Reward amount (in Peruvian soles). (DOCX)

S1 Text District descriptions. (DOCX)
