# Supplementary figures and images for: Incentivizing optimal risk map use for Triatoma infestans surveillance in urban environments
Source: PLOS Glob Public Health. 2022 Aug 3;2(8):e0000145. doi: 10.1371/journal.pgph.0000145 (PMC10021448; doi:10.1371/journal.pgph.0000145)

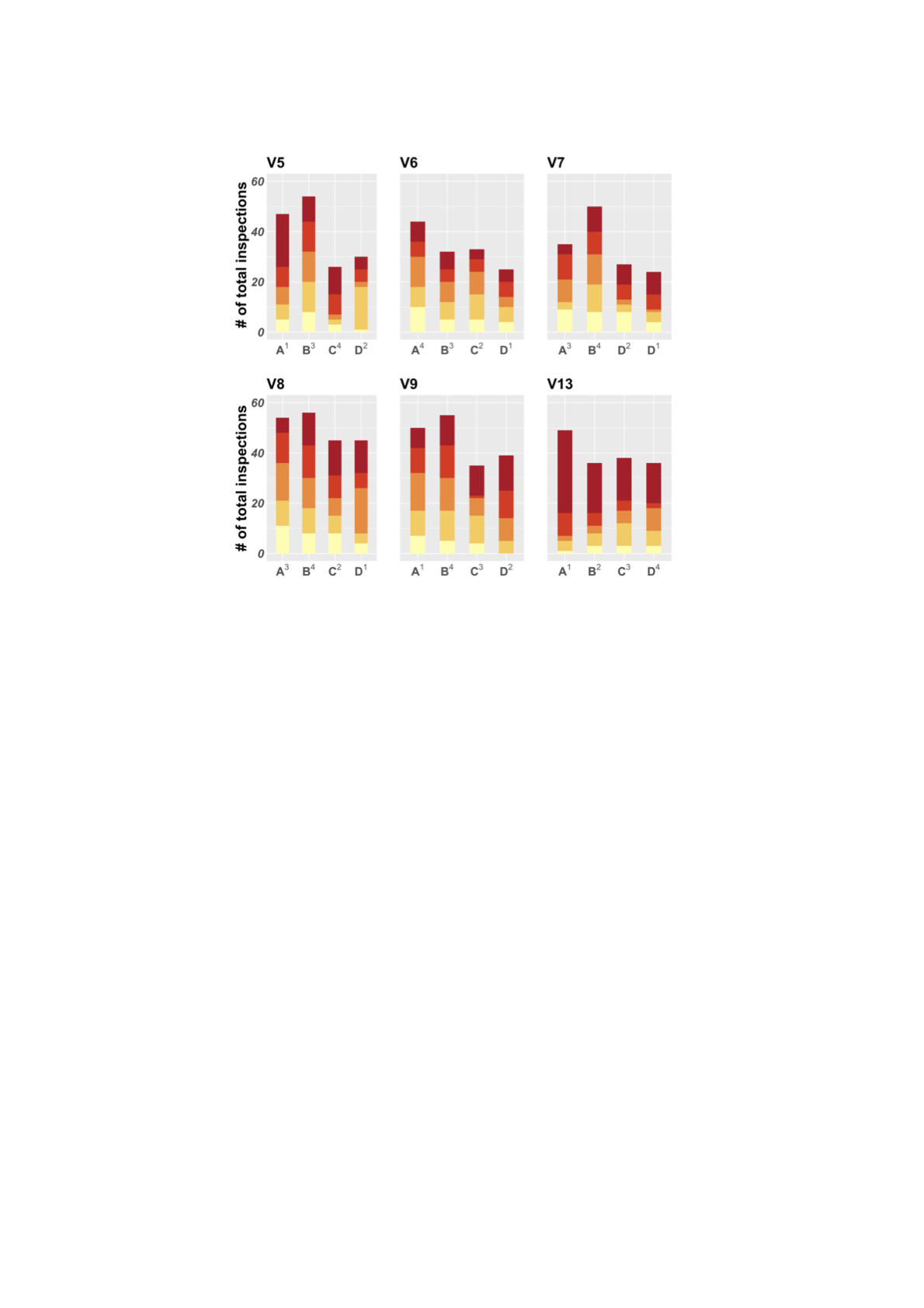

Supplement: S1 Fig — Each set of four bars represents one participant, and each bar a study arm (A-D). Colors are ordered by risk quintile, going from the lowest (light yellow, bottom) to the highest (dark red, top). Superscripts above each arm name (A-D) in the x axis text indicate arm order. (TIFF) [file pgph.0000145.s001.tiff]
