# Supplementary material for: Incentivizing optimal risk map use for Triatoma infestans surveillance in urban environments
Source: PLOS Glob Public Health. 2022 Aug 3;2(8):e0000145. doi: 10.1371/journal.pgph.0000145 (PMC10021448; doi:10.1371/journal.pgph.0000145)
Supplement: S1 Table — (DOCX) [file pgph.0000145.s002.docx]

|  | **Number of uninspected houses in the largest triangle** | | | | | | | | | |
| --- | --- | --- | --- | --- | --- | --- | --- | --- | --- | --- |
| **Participant** | **Socabaya trial** | | | | **Cayma trial** | | **JLByR trial** | | **Miraflores trial** | |
|  | A* | B* | C | D | A | B | A | B | A | B |
| V1 | - | - | - | - | - | - | - | - | 17 | 109 |
| V2 | - | - | - | - | - | - | - | - | 39 | 39 |
| V4 | - | - | - | - | 44 | 21 | 66 | 68 | 36 | 158 |
| V5 | 22 | 17 | 50 | 35 | 12 | 13 | 21 | 72 | 23 | 30 |
| V6 | 26 | 17 | 49 | 71 | 11 | 29 | 14 | 47 | 46 | 61 |
| V7 | 11 | 25 | 35 | 67 | 10 | 13 | - | - | - | - |
| V8 | 23 | 13 | 72 | 46 | - | - | 10 | 20 | 18 | 30 |
| V9 | 10 | 12 | 43 | 51 | 10 | 9 | 24 | 32 | 27 | 47 |
| V13 | 16 | 10 | 51 | 46 | 28 | 22 | 120 | 77 | 71 | 69 |
| V14 | - | - | - | - | - | - | - | - | 20 | 72 |
| MEAN | 18 | 15.7 | 50 | 52.7 | 19.2 | 17.8 | 42.5 | 60 | 33 | 68.3 |
| **S1 Table**. **Spatial coverage data by participant per trial and arm (A-D)**. Columns contain the number of uninspected houses in the largest triangle formed by connecting the vertices of all inspected houses following a week of inspections. Participants represented by ‘V[number].’ Dashes indicate that the participant did not participate in the trial. *Significant difference compared to control arm. Arm A: p < 0.01. Arm B: p < 0.001. | | | | | | | | | | |
