# Supplementary material for: Incentivizing optimal risk map use for Triatoma infestans surveillance in urban environments
Source: PLOS Glob Public Health. 2022 Aug 3;2(8):e0000145. doi: 10.1371/journal.pgph.0000145 (PMC10021448; doi:10.1371/journal.pgph.0000145)
Supplement: S2 Table — (DOCX) [file pgph.0000145.s003.docx]

| **Participant** | **Reward amount (in Peruvian soles^1^)** | | | | | | | | | |
| --- | --- | --- | --- | --- | --- | --- | --- | --- | --- | --- |
|  | Socabaya trial | | | | Cayma trial | | | | JLByR trial | |
|  | A  (Stochastic) | | B  (Stochastic) | | A**  (Fixed) | | B  (Stochastic) | | A  (Stochastic) | |
|  | *Risk info use* | ^*^*Spatial coverage* | *Risk info use* | *^*^Spatial coverage* | *Risk info use* | *Spatial coverage* | *Risk info use* | *Spatial coverage* | *Risk info use* | *Spatial coverage* |
| V1 | - | - | - | - | - | - | - | - | - | - |
| V2 | - | - | - | - | - | - | - | - | - | - |
| V4 | - | - | - | - | 15.9 | 18 | 12 | 42 | 0 | 0 |
| V5 | 4 | 84 | 12 | 70 | 15.2 | 64 | 0 | 58 | 0 | 23 |
| V6 | 12 | 156 | 6 | 54 | 12.5 | 66 | 12 | 24 | 10 | 26 |
| V7 | 4 | 152 | 6 | 60 | 17.3 | 68 | 0 | 62 | - | - |
| V8 | 0 | 152 | 12 | 70 | - | - | - | - | 10 | 34 |
| V9 | 12 | 128 | 6 | 66 | 19.4 | 78 | 12 | 78 | 0 | 21 |
| V13 | 0 | 76 | 12 | 50 | 16.4 | 44 | 6 | 40 | 20 | 0 |
| V14 | - | - | - | - | - | - | - | - | - | - |
| Mean | 5.33 | 124.7 | 9 | 64.3 | 16.1 | 56.3 | 7 | 50.7 | 6.7 | 17.3 |
| % salary  (monthly) | 0.45% | 10.6% | 0.8% | 5.5% | 1.4% | 3.4% | 0.6% | 4.3% | 0.6% | 1.4% |
| **S2 Table**. **Monetary rewards earned by participants**. Shown by trial, arm, and incentive type (risk information use and spatial coverage). Participants represented by ‘V[number].’ Dash indicates no participation in the trial. Results from the final trial (Miraflores) are in Table 8, as a different payout type was awarded. Final row shows payout as a percentage of the inspector’s monthly salary (approximately $350 USD). ^1^One sol ≈ $0.28USD. *Significant difference in spatial coverage compared to the control arm (p < 0.001). ** Risk information use in the fixed arm was significantly different than in the stochastic arm (Cayma trial only; p < 0.02). | | | | | | | | | | |
