# Supplementary material for: Incentivizing optimal risk map use for Triatoma infestans surveillance in urban environments
Source: PLOS Glob Public Health. 2022 Aug 3;2(8):e0000145. doi: 10.1371/journal.pgph.0000145 (PMC10021448; doi:10.1371/journal.pgph.0000145)
Supplement: S1 Text — (DOCX) [file pgph.0000145.s004.docx]

**S1. District descriptions**

**Socabaya*:*** The district of Socabaya is located in Southwest Arequipa. The district has a geographic area of 18.64 km^2^ and a growing population of 80,000 people. The insecticide spray portion of the vector control campaign took place in Socabaya in 2007, at which time *T. infestans* were detected in 9.8% of treated houses.

**Cayma:** The district of Cayma is located in the northern part of Arequipa, with a population of 103,458 [1,2] and a geographic area of 246 Km^2^. The insecticide spray phase of the *T. infestans* control campaign was carried out in Cayma in 2012, at which time 6.4% of targeted households in the district were infested (the district was sprayed in a focalized manner; not all the houses in the district were sprayed). Entomological surveillance has been more frequent in Cayma than in the Socabaya district.

**Jose Luis Bustamante y Rivero (JLByR):** The district is located in southeast Arequipa, and borders Socabaya. JLByR has a geographic area of 11.06 Km^2^ with a population of 76,410 people [3]. The insecticide application portion of the vector control campaign in the JLByR district took place in 2006, at which time 12.5% of houses were found to be infested with *T. infestans*.

**Miraflores:** Located in Northeast Arequipa, this district has an area of 28.7 km^2^ and a population of 104,068 inhabitants [4]. The insecticide application phase of the vector control campaign was carried out in Miraflores in 2011, at which time 2.2% of houses were found to be infested with *T. infestans*.

**References**

1. Comité Distrital de Seguridad Ciudadana CODISEC Cayma. Plan de acción distrital de seguridad ciudadana del distrito de Cayma. 2020.

2. Cori Mamani JA, Quispe Parizaca RY, Ruiz Martínez AS, Tafur Santillán CE, Yancachajlla Tito D. Planeamiento estratégico del distrito de Cayma. Tesis para obtener el grado de Magíster en administración estratégica de empresas, Pontificia Universidad Católica del Perú. 2018.

3. Municipal Distrital de Jose Luis Bustamante y Rivero. Plan de acción de seguridad ciudadana 2019. 2019.

4. Comité Distrital de Seguridad Ciudadana CODISEC Miraflores. Plan de acción distrital de seguridad ciudadana - Miraflores 2020. 2020 p. 80.
